# Inflammation from Sleep Fragmentation Starts in the Periphery Rather than Brain in Male Mice

**DOI:** 10.21203/rs.3.rs-2544592/v1

**Published:** 2023-02-13

**Authors:** Van Thuan Nguyen, Cameron J. Fields, Noah T. Ashley

**Affiliations:** Western Kentucky University; Western Kentucky University; Western Kentucky University

**Keywords:** Cytokines, Glucocorticoids, Inflammation, Interleukin-1, Interleukin- 6, Sleep Fragmentation, Tumor Necrosis Factor

## Abstract

Obstructive sleep apnea is increasing worldwide, leading to disordered sleep patterns and inflammatory responses in brain and peripheral tissues that predispose individuals to chronic disease. Pro-inflammatory cytokines activate the inflammatory response and are normally regulated by glucocorticoids secreted from adrenal glands. However, the temporal dynamics of inflammatory responses and hypothalamic-pituitary-adrenal (HPA) axis activation in relation to acute sleep fragmentation (ASF) are undescribed. Male C57BL/6J mice were exposed to ASF or control conditions (no ASF) over specified intervals (1, 2, 6, and 24 h) and cytokine gene expression (IL-1beta, TNF-alpha) in brain and peripheral tissues as well as serum glucocorticoid and interleukin-6 (IL-6) concentration were assessed. The HPA axis was rapidly activated, leading to elevated serum corticosterone from 1–24 h of ASF compared with controls. This activation was followed by elevated serum IL-6 concentration from 6–24 h of ASF. The tissue to first exhibit increased pro-inflammatory gene expression from ASF was heart (1 h of ASF). In contrast, pro-inflammatory gene expression was suppressed in hypothalamus after 1 h of ASF, but elevated after 6 h. Because the HPA axis was activated throughout ASF, this suggests that brain, but not peripheral, pro-inflammatory responses were rapidly inhibited by glucocorticoid immunosuppression.

## Introduction

The increased prevalence of obesity in the United States and other developed countries has drastically increased obstructive sleep apnea (OSA) diagnoses^1^. This condition leads to sleep fragmentation (SF), reduced blood oxygen saturation, increased daytime sleepiness, and the occurrence of inflammation in the brain and peripheral tissues^2,3^. While OSA is related to the development of chronic pathologies, such as metabolic ^4,5^ and cardiovascular diseases^6,7^ as well as neurological disorders^8^, the underlying mechanism of these studies is still unclear, although chronic inflammation is thought to play a large role in determining disease outcomes^2^.

Inflammation is a pervasive phenomenon that is typically triggered during the onset of infection, injury, or exposure to pollutants^9^. Sleep loss is also a potent inducer of inflammation^2,3,10^, but the mechanisms underlying this response are unclear. Sleep promotes the clearance of metabolic waste products, such as beta-amyloid protein^11–14^, and loss of sleep, in turn, reduces clearance, leading to a build-up of waste products that may trigger the immune system to produce inflammatory mediators, termed herein the “metabolic clearance hypothesis.” Based upon this possibility, the onset of inflammation from sleep loss could occur in the brain. The alternative hypothesis is that inflammation begins in the periphery with sympathetic afferents relaying this information to the brain, similar to a neuro-immune reflex to peripheral immune challenge^15^. As evidence, previous studies have shown that inhibition of the peripheral sympathetic nervous system (SNS) reduces inflammatory responses to SF in peripheral tissues^16,17^ and brain^18^.

Superimposed upon these inflammatory responses is activation of the hypothalamic-pituitary-adrenal (HPA) axis, which culminates in the release of glucocorticoids from the adrenal cortices and acts as a brake on immune function and inflammation^19^. Despite this long standing dogma, glucocorticoids can in some cases prime pro-inflammatory responses in the brain^20,21^. Interestingly, HPA axis activation occurs in tandem with pro-inflammatory responses to SF in mice^10,16,22,23^. Thus, it remains unresolved whether HPA activation provides negative feedback to inflammatory responses, temporarily potentiates responses, or has no effect.

The aim of this study was to evaluate the time course of peripheral and brain inflammatory responses in relation to HPA activation in male C57BL/6J mice exposed to acute SF. We predicted an initial increase in pro-inflammatory cytokine gene expression in brain that would be negatively regulated by a rise in serum corticosterone from HPA activation occurring later in the time course. We also predicted that neuroinflammatory responses would occur earlier than peripheral inflammatory responses, as suggested by the metabolic clearance hypothesis, which would represent the initial neuro-immune response to the build-up of metabolic waste products in brain. The alternative hypotheses are that the initiation of the inflammatory response from ASF occurs first in the periphery and then relayed to the brain through sympathetic afferents or that peripheral and brain inflammatory responses to sleep fragmentation occur independently from each other.

## Methods

### Animals.

Male adult C57BL/6J mice between 8–12 weeks of age were used in this study (*n*= 110; Jackson Laboratory, Bar Harbor, ME). Mice were given food and water ad *libitum* and housed under standard rodent colony conditions (lights on: 0800–2000 h, 21°C ± 1°C) at Western Kentucky University. Acute sleep fragmentation (ASF) experiments were performed using automated sleep fragmentation chambers (Lafayette Instrument Company; Lafayette, IN; model 80390) with a thin layer of corn bedding as previously described and each chamber contained no more than five mice^23^. These chambers ensure that mice are subjected to sleep fragmentation and not absolute sleep deprivation^16^. Mice were acclimated to the sleep fragmentation (SF) chambers for 48 h before the commencement of experiments to minimize carryover effects from the different cage environments^24^. This study was conducted under the approval of the Institutional Animal Care and Use Committee at Western Kentucky University (#19–11), and procedures followed the National Institutes of Health’s “Guide for the Use and Care of Laboratory Animals” and ARRIVE guidelines.

### Acute sleep fragmentation (Acute SF) and sample collection.

**Figure 1:**
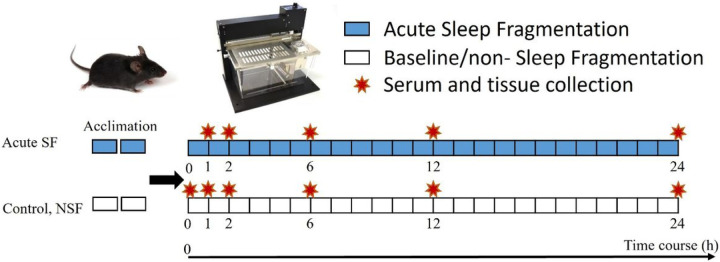
Experimental protocol for acute sleep fragmentation (ASF) time-course study. Mice were exposed to 1, 2, 6, 12, or 24 h of ASF, which involves a sweeping bar that moves horizontally across a modified cage every 120 sec. Controls (no sleep fragmentation (NSF)) mice experienced no bar movement.

Starting at 0800 (lights on), mice were exposed to 1, 2, 6, 12, or 24 h (n=110; all groups, *n* = 10) of ASF, which involves a sweeping bar that moves horizontally across the modified cage every 120 sec, simulating the rate of SF in patients with severe sleep apnea^25^ (Fig. 1). For the non-sleep fragmentation (NSF) control mice, subjects were housed in SF chambers, but no sweeping bar movements occurred. The NSF groups matched collection times of ASF mice (1, 2, 6, 12, or 24 h; all groups, *n* = 10). Both ASF and NSF groups were compared to a baseline group (time = 0) of mice collected at 0800 (*n* = 10).

#### Sample Collection.

After ASF or NSF treatments, mice were rapidly anesthetized using isoflurane induction (5%) and decapitated <3 min of initial handling for tissue gene expression studies and blood collection for measurement of CORT and interleukin-6 (IL-6) levels (see below). Trunk blood was collected from mice, kept on ice for <20 min, and spun at 3000×g for 30 min at 4°C. Serum was collected and stored at −80°C for corticosterone and IL-6 ELISA assays (see below). For gene expression analyses, three brain regions (prefrontal cortex (PFC), hypothalamus, and hippocampus), liver, spleen, heart, and epididymal white adipose tissue (EWAT) were dissected from mice and stored in RNAlater solution (ThermoScientific) in the freezer at −20°C. These particular brain regions and peripheral tissues were chosen because previous studies have demonstrated elevated pro-inflammatory gene expression from ASF^12, 18^. All tissue samples were stored at −20°C before RNA extraction.

### Corticosterone and Interleukin-6 ELISA.

Serum levels of corticosterone (n = 9–10/group) were measured using an ELISA kit (Catalogue number ADI-901–097, EnzoLife Sciences) which had a sensitivity of 26.99 pg/mL with cross-reactivity of <30% deoxycorticosterone and <2% progesterone. Samples were diluted 1:40 before running. The reaction was carried out in duplicate according to the kit instructions, and the average absorbance of the plate was determined using a plate reader (BioTek Synergy H1 Hybrid Reader). Average intra- and inter-assay variations were 2.85% and 2.65% respectively. IL-6 was measured in sera using ELISA MAX Deluxe kits (Catalogue number 431304; BioLegend, San Diego, CA). The assays were carried out according to the manufacturer’s instructions, and the average intra- and interassay variations were 8.24% and 7.43% respectively.

### Cytokine gene expression.

RNA was extracted from liver, spleen, epidydimal white adipose tissue (EWAT), as well as the prefrontal cortex, hippocampus, and hypothalamus from brain using a RNeasy mini kit (Qiagen). RNA was extracted from the heart using a RNeasy Fibrous Tissue mini kit (Qiagen). All extractions were performed following the manufacturer’s instructions. RNA concentrations were measured using a NanoDrop 2000 Spectrophotometer (Thermo Scientific). Total RNA was reverse transcribed using a high-capacity cDNA reverse transcription kit (ThermoFisher Scientific, Cat number: 4368813) according to the manufacturer’s instructions and used as a template for determining relative cytokine gene expression using an ABI 7300 RTPCR system. Tissues were analyzed with cytokine primers/probes (IL1β: Mm00434228, TNFα: Mm00443258; ThermoFisher Scientific). Assay probes were labeled with florescent marker 5-FAM and quencher MGB at the 5’ end and 3’ end, respectively, and VIC-labeled 18S primer/probe (primer-limited; 4319413E; ThermoFisher Scientific) was used as an endogenous control. A multiplex PCR assay which included the genes of interest, and the endogenous control was run simultaneously for each sample. Samples were run in duplicate and the fold change in mRNA level was calculated as the relative mRNA expression levels, 2^−ΔΔCt^. The cycle threshold (Ct) at which the fluorescence exceeded background levels was used to calculate ΔCt (Ct[target gene] – Ct[18S]). Each Ct value was normalized against the highest Ct value of a control sample (ΔΔCt), and then the negative value of this power to 2 (2 ^−ΔΔCt^) was used for mRNA expression analysis.

### Statistical Analysis.

Data are presented as mean (±SE). All statistical analyses were performed using GraphPad Prism (version 9.0). Two-way ANOVAs assessed the effect of sleep treatment (ASF or NSF), time (1h, 2h, 6h, 12h, 24h), and their interaction on mRNA expression of cytokines, serum CORT levels, and serum IL-6 concentration. One-way ANOVAs were used to assess whether ASF and NSF groups differed significantly from baseline levels (time 0h). Tukey’s HSD and Bonferroni multiple comparisons were used for post hoc analyses for one-way ANOVA and two-way ANOVA, respectively. p < 0.05 was considered statistically significant.

## Results

### Serum Corticosterone Concentration.

**Figure 2:**
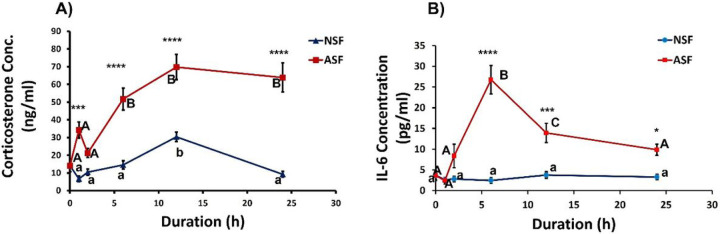
Duration of acute sleep fragmentation (ASF) alters baseline glucocorticoid and IL-6 levels in serum. A) Corticosterone (cort) concentration in male mice subjected to acute SF (0, 1, 2, 6, 12, and 24 h of ASF or no SF (NSF)). Samples sizes are *n*= 9–10 per group. B) IL-6 levels in male mice subjected to acute SF (0, 1, 2, 6, 12, and 24 h of ASF or no SF (NSF)). Samples sizes are *N* = 9–10/group. Significant effect of ASF (*** and **** denote p < 0.001 and 0.0001, respectively) relative to NSF at each time point was determined by two-way ANOVA followed by Bonferroni multiple comparisons post hoc tests. Differing lowercase and upper-case letters denote p < 0.05 for NSF and ASF groups, respectively and were analyzed using a one-way ANOVA and Tukey’s HSD post hoc tests. Bar plots shown as means ± 1 SE and *p* was set at 0.05 for statistical significance.

Serum corticosterone levels were significantly affected by sleep treatment (*F*_1, 86_ = 131.2, *p* < 0.0001, Fig. 2A), time (*F*_4, 86_ = 16.71, *p* < 0.0001) and their interaction (*F*_4,86_ = 5.917, *p* = 0.0003). Corticosterone levels were significantly higher at 1, 6, 12, and 24h (Bonferroni post hoc test, *p* = 0.0004, < 0.0001, < 0.0001, < 0.0001, respectively) among ASF mice compared with NSF mice. Serum corticosterone concentration of NSF mice (*F*_5,50_ = 12.84, *p* < 0.0001) increased from 0h to 12h and decreased from 12h to 24h, respectively (12h time point, Tukey’s HSD post hoc, *p* = 0.0002 and < 0.0001 compared to other points). ASF mice significantly increased serum corticosterone levels from 0h starting at 6h (*F*_5,52_ = 15.02, *p* < 0.0001; Tukey’s HSD post hoc, *p* = 0.0005), and remained elevated above baseline at 12 h (*p* = < 0.0001) and 24 h (*p* = < 0.0001) following ASF.

### Serum Interleukin-6 (IL-6) Concentration.

Sleep treatment, time, and their interaction significantly affected serum IL-6 levels (*F*_1, 88_ = 73.19, *p* < 0.0001, *F*_4, 88_ = 13.73, *p* < 0.0001, *F*_4,88_ = 14.27, *p* < 0.0001, respectively; Fig. 2B). Specifically, ASF mice exhibited increased IL-6 levels compared with NSF mice at 6, 12, and 24h after treatment (Bonferroni post hoc test, *p* < 0.0001, *p* = 0.0003, *p* < 0.0371, respectively). NSF mice displayed stable IL-6 levels (*F*_5,53_ = 0.6383, *p* = 0.6714) around 3–4 pg/ml while ASF mice had a rapid increase at 6 h of ASF (*F*_5,53_ = 16.24, *p* < 0.0001; 6h, Tukey’s HSD post hoc, *p* < 0.0001), followed by a decrease at 12 and 24 h (Tukey’s HSD post hoc, *p* = 0.0016, *p* < 0.0001, respectively) of ASF.

### Peripheral Responses.

#### Spleen.

**Figure 3:**
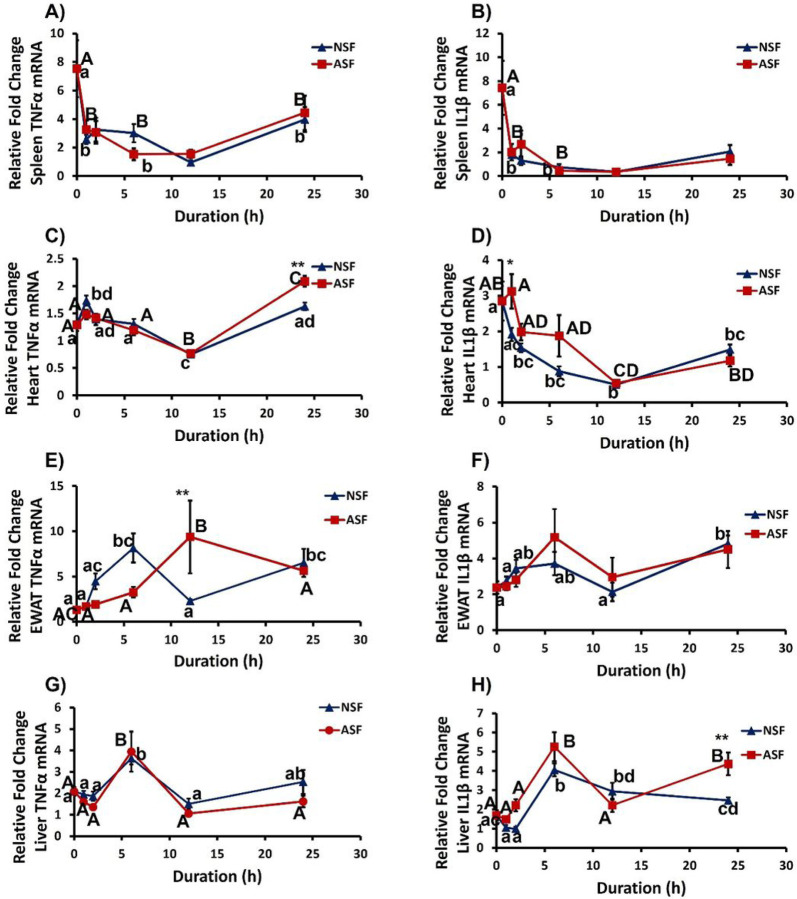
Effects of sleep fragmentation, time, and their interaction on TNFα and Il-1β gene expression in peripheral tissues. Panels show TNFα and Il-1β gene expression in spleen (A, B), heart (C, D), epididymal white adipose tissue (EWAT; E, F) and liver (G, H) (n = 8–10/group, time-course: 0, 1, 2, 6, 12, 24h). Significant effect of sleep treatment (** denotes p < 0.01) at each time point was determined by two-way ANOVA followed by Bonferroni multiple comparisons post hoc test. Differing lowercase and upper-case letters denote *p*< 0.05 across different time points for NSF and ASF, respectively and were analyzed using a one-way ANOVA and Tukey’s HSD post hoc tests. Bar plots shown as means ± 1 SE and *p* was set at 0.05 for statistical significance.

ASF had no effect on TNFα (*F*_1, 84_ = 0.001368, *p* = 0.9706) or IL1β (*F*_1,84_ = 0.1895, *p* = 0.6645) gene expression in spleen (Fig. 3A–B). However, TNFα (*F*_4,84_ = 22.17, *p* = 0.0012) and IL1β expression (*F*_4,84_ = 4.822, *p* = 0.0015) were affected by time compared with NSF mice, but there were no significant interaction effects (TNFα: *F*_4,84_ = 0.8545, *p* = 0.4948; IL1β: *F*_4,84_ = 1.066, *p* = 0.3785).

#### Heart.

Sleep treatment had no overall effect upon TNFα expression in heart (*F*_1,83_ = 0.2545, *p* = 0.6152; Fig. 3C). There were significant effects of time (*F*_4,83_ = 47.06, *p* < 0.0001) and the interaction between sleep treatment and time (*F*_4,83_ = 5.024, *p* = 0.0011). More specifically, at the 24-h time point, ASF mice exhibited increased TNFα expression compared with NSF mice (Bonferroni post hoc test, *p* = 0.0014). In contrast, there was a significant effect of sleep treatment (*F*_1,86_ = 7.045, *p* = 0.0095, Fig. 3D), time (*F*_4,86_ = 13.66, *p* < 0.0001), and their interaction (F_4,86_ = 2.509, *p* = 0.0477) upon IL1β expression in heart. At the 1h time point, ASF mice increased cardiac IL1β expression relative to NSF mice (Bonferroni post hoc test, *p* = 0.0136).

#### EWAT.

Sleep treatment had no overall effect on TNFα (*F*_1, 83_ = 0.06913, *p* = 0.7933) or IL1β (*F*_1, 82_ = 0.1820, *p* = 0.6708) gene expression in EWAT (Fig. 3E–F). However, time affected both TNFα (*F*_4, 83_ = 3.492, *p* = 0.0110) and IL1β (*F*_4, 82_ = 3.340, *p* = 0.0138), but there was only a significant interaction effect for TNFα gene expression (TNFα: *F*_4, 83_ = 4.754, *p* = 0.0017; IL1β: *F*_4, 82_ = 0.6524, *p* = 0.6268). At the 12h time point, ASF mice has significantly higher TNFα expression in EWAT compared with NSF mice (Bonferroni post hoc test, *p* = 0.0047).

#### Liver.

There was neither a significant effect of sleep treatment nor an interaction effect on hepatic TNFα expression (sleep treatment, *F*_1, 82_ = 2.254, *p* = 0.1371; interaction: *F*_4, 82_ = 0.5843, *p* = 0.6749), but there was a significant effect of time on TNFα expression in liver (*F*_4, 82_ = 11.67, *p* < 0.0001; Fig. 3G). Sleep treatment, time, and their interaction had a significant effect on IL1β gene expression in liver (sleep treatment: *F*_1, 81_ = 10.81, *p* = 0.0015; time: *F*_4, 81_ = 25.37, *p* < 0.0001; interaction: *F*_4, 81_ = 3.332, *p* = 0.0141). More specifically, IL1β gene expression was significantly higher in ASF mice compared with NSF mice at the 24h time point (Bonferroni post hoc test, *p* < 0.05; Fig. 3H).

### Brain Responses.

#### Hippocampus.

**Figure 4:**
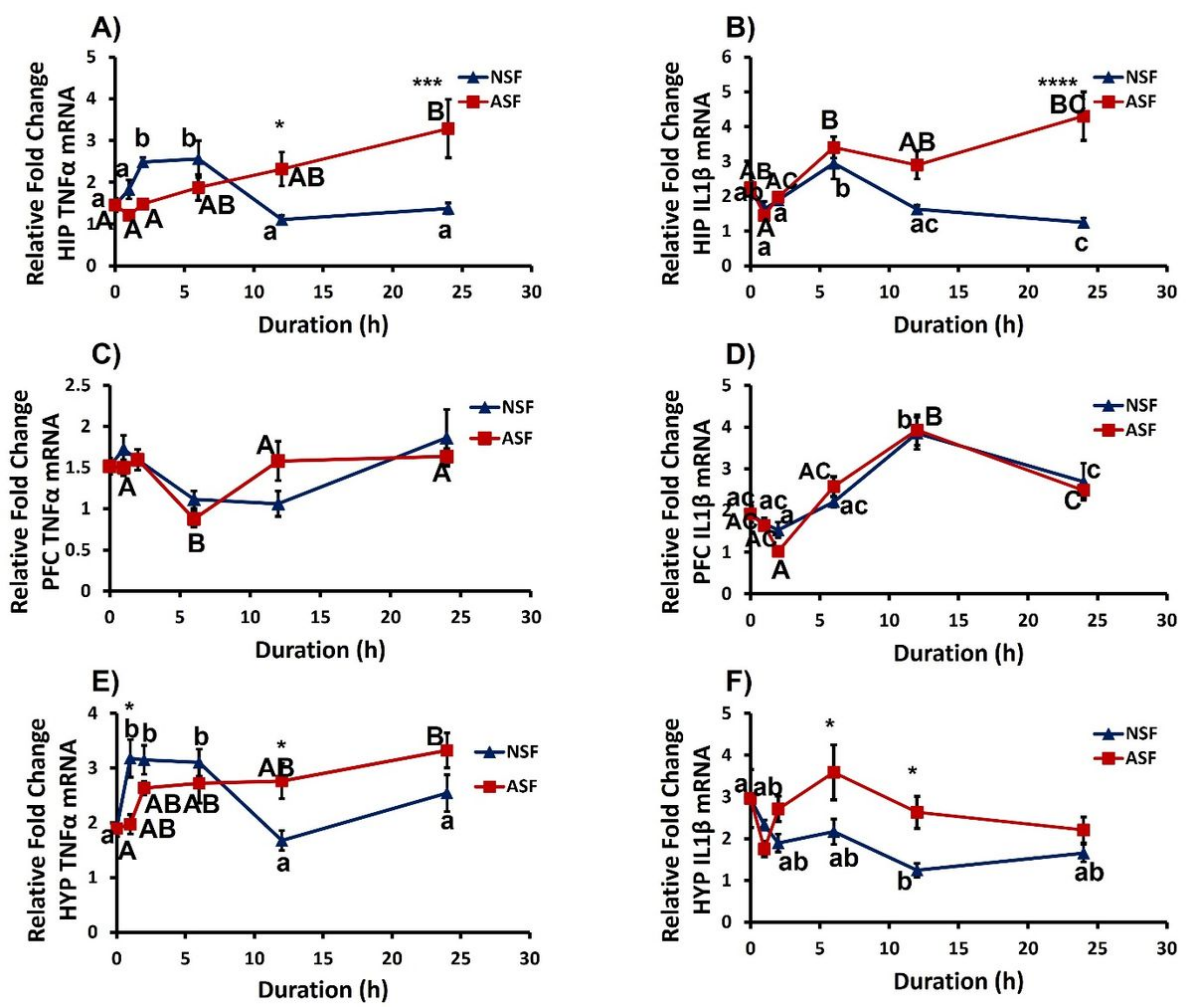
Effects of sleep fragmentation, time, and their interaction on TNFα and Il-1β gene expression in brain, PFC (A, B), HIP (C, D), and HYP (E, F) (n = 9–10/group, Acute SF time-course: 0, 1, 2, 6, 12, 24h). Significant effect of SF (*, ** and *** denote p < 0.05, 0.01, and 0.001, respectively) was determined by two-way ANOVA followed by Bonferroni multiple comparisons post hoc test. Differing lowercase and upper-case letters denote p < 0.05 across different time points for NSF and ASF, respectively and were analyzed using a one-way ANOVA and Tukey’s HSD post hoc tests. Bar plots shown as means ± 1 SE and p was set at 0.05 for statistical significance. HIP: Hippocampus; PCF: prefrontal cortex; HYP: Hypothalamus.

Although sleep treatment or time course did not affect hippocampal TNFα (Acute SF: *F*_1, 83_ = 0.6730, *p* = 0.4144; time course: *F*_4, 83_ = 2.327, *p* = 0.0629) gene expression in the hippocampus, the interaction effect was significant (*F*_4, 83_ = 8.365, *p* < 0.0001). ASF mice exhibited increased TNFα expression in hippocampus compared with NSF at 12h (Bonferroni post hoc test, *p* = 0.0389) and 24h (Bonferroni post hoc test, *p* < 0.0003) time points (Fig. 4A). Additionally, sleep treatment, time, and their interaction had a significant effect on hippocampal IL1β expression (sleep treatment: *F*_1, 88_ = 16.94, *p* < 0.0001; time: *F*_4, 88_ = 6.622, *p* = 0.0001; interaction: *F*_4, 88_ = 6.650, *p* = 0.0001; Fig. 4B).

#### Prefrontal cortex (PFC).

Sleep treatment had no effect on TNFα or IL1β (TNFα: *F*_1, 86_ = 0.08523, *p* = 0.7710; IL1β: *F*_1, 85_ = 0.1395, *p* = 0.7097). However, time had a significant effect on TNFα and IL1β (TNFα: *F*_4, 86_ = 5.465, *p* = 0.0006; IL1β: *F*_4, 85_ = 30.24, *p* < 0.0001) gene expression (Fig. 4C–D), but there were no significant interaction effects.

#### Hypothalamus.

Sleep treatment had no overall effect upon hypothalamic TNFα expression (*F*_1, 85_ = 0.07240, *p* = 0.7885). However, there were significant effects of time (*F*_4, 85_ = 2.535, *p* = 0.0460) and the interaction between sleep treatment and time (*F*_4, 85_ = 6.042, *p* = 0.0003; Fig. 4E). In hypothalamus, there were significant effects of sleep treatment, time, and their interaction on IL1β gene expression (sleep treatment: *F*_4, 84_ = 12.21, *p* = 0.0008; time: *F*_4, 84_ = 3.118, *p* = 0.0192; interaction: *F*_4, 84_ = 3.173, *p* = 0.0177). More specifically, ASF mice exhibited significantly lower IL1β gene expression level at 1h (Bonferroni post hoc test, *p* = 0.0115) and significantly higher levels at 12h (Bonferroni post hoc test, *p* = 0.0289) compared with NSF mice (Fig. 4F).

## Discussion

Sleep loss induces an inflammatory response in various tissues of the body^10^, but the temporal dynamics of inflammatory responses are unclear. Results from this time-course study fail to support the metabolic clearance hypothesis that inflammation from acute sleep fragmentation (ASF) begins in the brain. Instead, elevated pro-inflammatory gene expression was first detected in heart tissue 1 h after ASF. The first brain region measured that exhibited neuroinflammation was the hypothalamus after 6 h of ASF. However, there was suppression of pro-inflammatory gene expression at 1 h of ASF. These findings support the alternative hypothesis that peripheral inflammation from sleep fragmentation occurs first in the periphery, specifically the heart, rather than brain. Previous studies have documented increased pro-inflammatory gene expression in cardiac tissue following short-term^10,16^ and chronic sleep fragmentation^16,26^. We surmise that pro-inflammatory cytokines are elevated rapidly in heart due to increased SNS activity from sleep fragmentation^23,27,28^. Whether the heart directly relays inflammatory “information” to brain through sympathetic afferents or whether neuroinflammation is simply a slower, but independent, process requires further study. In addition, this study only measured select regions in brain and several organs/tissues in the periphery. Therefore, it is possible that other tissues that were not measured may have different temporal responses to ASF.

The HPA axis was rapidly activated from ASF as measured by increased corticosterone concentration in serum, which is a consistent finding from previous studies^10,18,22,23^. NSF mice exhibited a well-known diurnal rhythm in circulating corticosterone level with peak levels occurring at the onset of nocturnal activity^29^. Among ASF mice, serum corticosterone increased rapidly from 0h to 1h and remained elevated for the entire time course compared with NSF mice. As stated above, at 1h of ASF, there was suppression of TNF-α expression in hypothalamus, but an elevation of IL-1β expression in heart compared with NSF controls. These findings imply that the increased serum corticosterone concentration at this 1h time point may be rapidly suppressing an inflammatory response in brain, such as hypothalamus, but has no suppressive effect or even possibly a pro-inflammatory effect in the periphery (Table 1).

ASF also elevated serum IL-6 levels concomitant with increased serum corticosterone levels, with IL-6 levels peaking after 6 h but falling thereafter. Previous studies employing various forms of sleep deprivation on mice and humans have described elevated serum IL-6 concentration^30,31^. IL-6 acts pleiotropically through multiple pro- and anti-inflammatory pathways^32^, and glucocorticoids can alter the balance of these pathways through interfering with the expression of the suppressor of cytokine signalling 3 (SOCS3) feedback inhibitor^33^, as well as repressing the transcriptional activation of nuclear factor-kappa B (NF-κB)^34^. Whether elevated serum corticosterone concentration provided negative feedback to decrease serum IL-6 levels 12 and 24 h after ASF (relative to 6 h) will require additional investigation. In addition, it is also unknown whether IL-6 reciprocally activated the HPA axis as is often the case for bi-directional neuroendocrine-immune interactions^35^. Moreover, studies that manipulate glucocorticoid action either through adrenalectomy/hormone replacement experiments or pharmacological approaches that inhibit glucorticoid synthesis and/or receptor binding are needed to pinpoint the precise modulatory effects of glucocorticoids. It is also possible that activation of the SNS from acute sleep fragmentation through release of norepinephrine/epinephrine from adrenal medullae could promote a pro-inflammatory effect in the periphery that is independent of glucocorticoid effects. As evidence, suppression of the SNS using chemical sympathectomy alleviates inflammatory responses from acute and chronic sleep fragmentation in peripheral tissues^16^ as well as brain^18^.

The findings indicate that pro-inflammatory responses to acute sleep fragmentation are tissue-specific, which is consistent with previous studies^16–18, 23^. ASF increased TNFα expression in heart and EWAT after exposure for 12 and 24 h, respectively, but there was no effect in liver or spleen. In addition, TNFα expression from ASF occurred earliest in EWAT at 12h while others were at 24h (Fig. 3A, C, E; Table 1). This finding is consistent with previous studies, supporting the role of EWAT in the pro-inflammatory response to acute sleep fragmentation^22,23^.

In brain, ASF significantly increased IL-1β expression in hypothalamus and hippocampus at 6h, 12h, and 24h time points, respectively, but did not affect IL-1β in prefrontal cortex (Table 1). The brain responses for IL-1β gene expression were similar compared to TNFα but earlier. These results are a departure from previous studies that have shown elevated pro-inflammatory gene expression in hypothalamus, hippocampus, and pre-frontal cortex after 24-h of sleep fragmentation among female C57BL/6J mice^17,18^, but very few effects observed among males^10,23^, although there was a non-significant trend for increased pro-inflammatory gene expression in hippocampus in one study^12^. Reasons for this discrepancy are unclear, but could involve differences in how hippocampus was extracted from the brain in these different studies (whole vs. partial dissection).

## Conclusions

Sleep fragmentation and other forms of perturbed sleep promote an inflammatory environment that predisposes individuals towards the development of chronic disease^2,3^. However, the mechanisms that lead to the onset of inflammation from sleep fragmentation are poorly understood. Does inflammation begin in the brain or the periphery? In this time-course study, we provide evidence that the heart is the first organ to produce elevated pro-inflammatory gene expression, which suggests that inflammation from sleep fragmentation is rapidly initiated in the periphery. Because glucocorticoids are also elevated during sleep fragmentation, our findings imply that glucocorticoids may rapidly suppress inflammatory responses in certain regions of the brain, like hypothalamus, but not in peripheral tissues, such as heart and white adipose tissue. Instead, the rapid activation of the sympathetic nervous system from sleep fragmentation is most likely promoting an inflammatory environment in peripheral tissues, while possibly overriding negative feedback from glucocorticoids, albeit further study is required. Understanding the time course of inflammatory responses to sleep fragmentation could lead to new therapeutic options for patients suffering from disrupted sleep to prevent the development of cardiovascular and metabolic syndromes.

## Figures and Tables

**Table 1.** Timing of Pro-Inflammatory Cytokine mRNA Expression Levels from ASF or NSF. Summary of main effects of ASF, time, and their interaction on TNFα and IL1β cytokine gene expression levels in brain and peripheral tissues. epididymal white adipose tissue: EWAT, SF: sleep fragmentation.

Elevation in pro-inflammatory gene expression (TNF-α ⬆, IL-1β ⬆)					
Tissue	Acute SF time Points (course)				
	1h	2h	6h	12h	24h
**Liver**					⬆
**Heart**	⬆				⬆
**Spleen**				⬆	
**EWAT**					
**Prefrontal cortex**					
**Hypothalamus**	⬇		⬆	⬆ ⬆	⬆
**Hippocampus**				⬆	⬆ ⬆

## Data Availability

Datasets are available at Dryad https://datadryad.org/stash/share/3DqU5l67tr4V45PSzpnJmnAT3wLuQNBQo0oikQXIMk4.
